# Identifying risks areas related to medication administrations - text mining analysis using free-text descriptions of incident reports

**DOI:** 10.1186/s12913-019-4597-9

**Published:** 2019-11-04

**Authors:** Marja Härkänen, Jussi Paananen, Trevor Murrells, Anne Marie Rafferty, Bryony Dean Franklin

**Affiliations:** 1 Department of Nursing Science, University of Eastern Finland, Yliopistoranta 1c, Kuopio, Finland; 2 Institute of Biomedicine, University of Eastern Finland, Yliopistoranta 1c, Kuopio, Finland; 3 Florence Nightingale Faculty of Nursing, Midwifery & Palliative Care, King’s College London, James Clerk Maxwell Building, 57 Waterloo Road, London, SE1 8WA UK; 4 Centre for Medication Safety and Service Quality, Imperial College Healthcare NHS Trust, Charing Cross Hospital, Fulham Palace Road, / UCL School of Pharmacy, London, UK

**Keywords:** Incident reports, Medication administration, Text mining, Clustering, Risk

## Abstract

**Background:**

Some medications carry increased risk of patient harm when they are given in error. In incident reports, names of the medications that are involved in errors could be found written both in a specific medication field and/or within the free text description of the incident. Analysing only the names of the medications implicated in a specific unstructured medication field does not give information of the associated factors and risk areas, but when analysing unstructured free text descriptions, the information about the medication involved and associated risk factors may be buried within other non-relevant text. Thus, the aim of this study was to extract medication names most commonly used in free text descriptions of medication administration incident reports to identify terms most frequently associated with risk for each of these medications using text mining.

**Method:**

Free text descriptions of medication administration incidents (*n* = 72,390) reported in 2016 to the National Reporting and Learning System for England and Wales were analysed using SAS® Text miner. Analysis included text parsing and filtering free text to identify most commonly mentioned medications, followed by concept linking, and clustering to identify terms associated with commonly mentioned medications and the associated risk areas.

**Results:**

The following risk areas related to medications were identified: 1. Allergic reactions to antibacterial drugs, 2. Intravenous administration of antibacterial drugs, 3. Fentanyl patches, 4. Checking and documenting of analgesic doses, 5. Checking doses of anticoagulants, 6. Insulin doses and blood glucose, 7. Administration of intravenous infusions.

**Conclusions:**

Interventions to increase medication administration safety should focus on checking patient allergies and medication doses, especially for intravenous and transdermal medications. High-risk medications include insulin, analgesics, antibacterial drugs, anticoagulants, and potassium chloride. Text mining may be useful for analysing large free text datasets and should be developed further.

## Background

Pharmacotherapy is an essential part of medical care for most patients [1]. Some medications carry increased risk of substantial patient harm when given in error, and are sometimes referred to as ‘high-alert’ medications. According to the US Institute for Safe Medication Practices (ISMP), in acute care settings, these drugs include anaesthetics, anti-arrhythmics, anti-thrombotics, chemotherapeutic medications, dialysis solutions, epidural or intrathecal medications, insulin, narcotics/opioids, and parenteral nutrition [2]. The high risk drug list developed by the National Patient Safety Agency (NPSA) for England and Wales includes methotrexate, diamorphine /morphine injections, low molecular weight heparins, anticoagulants, insulin, lithium, midazolam injection, opioids, injectable and liquid medicines [3]. In addition, a systematic review revealed that almost half of all serious medication errors were caused by seven drugs /drug classes: methotrexate, warfarin, nonsteroidal anti-inflammatory drugs (NSAIDS), digoxin, opioids, aspirin, and beta-blockers [4]. Not every incident causes serious or life-threatening harm, but they may still result in additional work, extra costs, discomfort and extended hospital stays. Thus, it is important to understand the type of medication implicated in medication administration incidents.

Incident reports are gathered voluntarily or mandatorily in many health care organisations worldwide. Incident reports are difficult to use in a systematic way because of the nature and limitations of reports, such as missing and other invalid data. It is therefore important to identify innovative ways of learning from them. The information in incident reports can be both structured, and unstructured (i.e. as free text descriptions). Free text information includes valuable data about factors related to incidents that may remain hidden if solely relying on structured information [5]. Such information can be extracted with advanced informatics techniques [6] particularly when datasets are too large for manual analysis.

Text mining employs multiple techniques from different fields, including machine learning, natural language processing (NLP), biostatistics, information technology, and pattern recognition [7]. It attempts to discover patterns in unstructured data using indexing, searching, NLP analyses and language synthesis [8], to find new meanings hidden in the text [7]. It is therefore possible to analyse words, clusters of words, or whole documents to find associations and similarities and explore how these entities are related to other variables [9]. As more and more incidence reports are being generated and hospital information systems integrated, there is so much data that manual inspection of this data is not feasible and text mining is the way to analyse these large masses of information in a data-driven way. Text mining allows using all this information to answer a wide variety of questions rapidly, as well as enables developing automated monitoring systems to proactively react to changes in trends in incidence reports.

In previous studies, free text information relating to medications has been extracted by text mining from clinical notes [6, 10], narrative discharge summaries [1, 11], or from free-text prescriptions [12]. These studies have mostly focused on the identification of textual expressions that refer to drug usage and characteristics (medication dose, mode of administration, frequency or duration) rather than trying to convert them into a structured form that can be then used directly for data analytics [12].

Names of medications involved in errors are usually written both in a specific medication field and/or within the free text description of the incident reports. Analysing only the names of the medications specified in the medication field does not give information about any associated contributing factors or risk areas. In turn, medication-related information in narrative free text can be buried within other non-relevant text [1]. Thus, the aim of this study was to explore the use of text mining methodology to extract the names of medications most commonly mentioned in free text descriptions of medication administration incident reports and identify terms most frequently associated with risk for each of these medications.

## Methods

### Design and setting

This was a retrospective study using information of medication administration incidents reported in England and Wales.

### Description of the data

The data comprised medication administration incidents (*n* = 72,390) sent to the National Reporting & Learning System (NRLS) database as having been reported by acute care hospitals in England and Wales between 1 January and 31 December 2016. This analysis focuses on the free text descriptions of the incidents, but draws in some categorical data where necessary.

### Data analysis

**Fig. 1:**
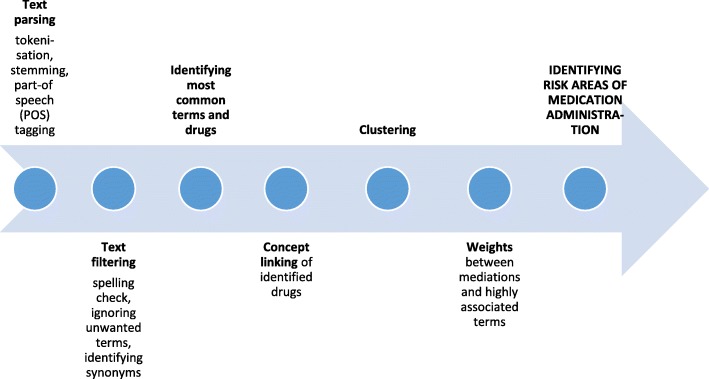
Analysis process of medication administration incident reports’ free text descriptions

Text data (Excel file) was first converted into SAS format for importing into Text Miner where the algorithms would be applied. The SAS® Enterprise Miner 13.2 and its Text Miner tool, and descriptive modelling with a ‘bag-of-words method’ were used to count words in the text and to understand how these words related to each other. Analysis included multiple steps as described in Fig. 1.

#### Text parsing and filtering

SAS® Text Miner automatically processes the data using ‘text parsing’ node of the programme i.e. converting unstructured text into a structured form suitable for data mining. Text parsing includes tokenisation (breaking text into words / terms), stemming (which chops off the end of words reducing words to their stem or root forms), and part-of text tagging (for each word, the algorithm decides whether it is a noun, verb, adjective, adverb, preposition and so on). ‘Text filtering’ is then used to reduce the total number of parsed terms, and check the spellings. The English language was used for parsing and filtering the text. A SAS Text Miner stop list (a list of all of the possibly irrelevant words) was used, so parts of the text including auxiliary verbs, conjunctions, possessive pronoun, interjections, numbers, participles, prepositions, and pronouns were ignored. The method is described in more detail elsewhere [13]. Synonyms were combined manually using an interactive filter viewer. Unwanted terms (such as most abbreviations) were excluded, as well as terms occurring in fewer than in ten reports. Most commonly cited drugs described in the free text descriptions were identified manually using an interactive filter viewer and its list of the most common terms in the data.

#### Concept linking

Further analysis included ‘concept linking’ to identify other terms that are highly associated with a selected term. The selected term is shown at the centre of a link diagram, and the terms that circle this are those that occur together most often with that central term [13, 14]. The strength of association between terms in a corpus of documents is calculated using the binomial distribution [15]. Concept linking was conducted for the most commonly cited drugs in the free text descriptions analysed.

#### Clustering

Cluster analysis or ‘clustering’ is a process of grouping a set of objects with similar content into the same cluster, so that using a distance metric like similarity of incidence reports, members of each group are as close as possible to one another and different groups are as far apart as possible. Once the clusters are determined, examining the words that occur in the cluster can reveal the focus of the cluster. Forming clusters within a collection of documents can facilitate understanding of and summarize the collection without reading every document (or in this case, incident report) as clusters can reveal the central themes and key concepts [14].

Clustering was carried out using singular value decomposition to transform the original weighted, term-document frequency matrix into a dense but low dimensional representation [14], which can improve the quality of clustering [5]. The expectation-maximization algorithm is the extension of the k-mean algorithm [5]. The content of clusters are usually various, thus human investigation and interpretation is needed [16]. In this study, different combinations of clusters were tested and the final number of clusters chosen based on subjective judgement and using root mean square standard deviation (RMSSD) values for each cluster group. RMSSD values were computed for every cluster for testing the goodness of fit or average distance between the observations in clusters. A small RMSSD value indicates that clusters are well defined and that documents within the clusters are very similar to each other. There is no established criterion for choosing a cut-off value for RMSSD, so it is a subjective decision [5]. The final number of clusters was set to a maximum of 20 (based on the lowest RMSSD values, and since setting the maximum level of clusters up to 25 did not produce any new clusters), the number of descriptive terms was set to 10.

#### Weights between medications and highly associated terms

Weights among identified medications (based on clustering), and terms highly associated with these medications (based on concept linking and clustering) were analysed using the document search field in the interactive filter viewer of SAS® Text Miner. The matching documents were retrieved using the vector space model. Weight is highest when the term occurs many times within a smaller number of documents and lowest when the term occurs in almost all documents [17].

## Results

### Data characteristics

The majority of the identified medication administration incidents were reported as not causing patient harm (86.3%, *n* = 62,461). The most common error types were omission (27.4%, *n* = 19,815), other (17.3%, *n* = 12,528), and wrong frequency (9.6%, *n* = 6975). The majority (65.1%, *n* = 47,149) of incidents occurred on wards (Table 1).

**Table 1:** Characteristics of medication administration incidents (*n* = 72,390)

Characteristics	No.	%
Severity		
No harm	62,461	86.3
Low	9,147	12.6
Moderate	708	1.0
Death	46	0.1
Error type		
Omitted medicine / ingredient	19,815	27.4
Other	12,528	17.3
Wrong frequency	6,975	9.6
Wrong drug	5,494	7.6
Wrong /unclear dose or strength	5,187	7.2
Wrong quantity	4,415	6.1
Mismatching between patient and medicine	3,437	4.7
Wrong storage	2,091	2.9
Wrong method of preparation / supply	2,001	2.8
Wrong route	1,715	2.4
Adverse drug reaction (when used as intended)	1,584	2.2
Wrong / omitted / passed expiry date	1,458	2.0
Wrong formulation	1,256	1.7
Patient allergic to treatment	1,248	1.7
Unknown	1,131	1.6
Contra-indication to the use of the medicine in relation to drugs or conditions	1,079	1.5
Wrong / transposed / omitted medicine label	505	0.7
Wrong /omitted verbal patient directions	406	0.6
Wrong / omitted patient information leaflet	65	0.1
Location		
Ward	47,149	65.1
Intensive care unit / high dependency unit	5,034	7.0
Operating theatre	1,480	2.0
Pharmacy	1,423	2.0
Hospital buildings (inside)	1,299	1.8
Other	944	1.4
Radiology	449	0.6
Recovery room	315	0.4
Anaesthetic room	237	0.3
Missing information	14,060	19.4
TOTAL	72,390	100

Based on the data field for the medication involved (approved drug name), the most common medications involved were insulin (*n* = 2577), morphine (*n* = 2541), paracetamol (*n* = 2155), sodium / sodium chloride (*n* = 1755), oxycodone (*n* = 1429), co-amoxiclav (*n* = 1039) and potassium / potassium chloride (*n* = 1032) (Table 2).

**Table 2:** Most common drugs described in categorical field (approved drug name) of incident reports (*n* = 72,390)

	Drug	Freq.
1.	insulin	2577
2.	morphine	2541
3.	paracetamol	2155
4.	sodium / sodium chloride	1755
5.	oxycodone	1429
6.	co-amoxiclav	1039
7.	potassium / potassium chloride	1032
8.	unknown	1019
9.	chloride	974
10.	enoxaparin	970
11.	fentanyl	880
12.	gentamicin	853
13.	dalteparin	852
14.	sulphate	783
15.	furosemide	652
16.	Tazocin [piperacillin / tezobactam]	631
17.	warfarin	542
18.	glucose	521
19.	heparin	419
20.	vancomycin	413
21.	aspirin	353
22.	tramadol	350
23.	dexamethasone	338
24.	flucloxacillin	336
25.	ibuprofen	324
26.	codeine	291
27.	midazolam	287
28.	benzylpenicillin	284
29.	saline	271
30.	nutrition	267

### Medications related to incidents and highly associated terms

The most common medications or medication types that were described in the reports’ free text were insulin (*n* = 10,086), antibiotic (*n* = 6280), paracetamol (*n* = 5449), and morphine (*n* = 4194) (Table 3). The most common terms associated with words describing **antibiotics** (antibiotic, gentamicin, amoxicillin, penicillin, intravenous antibiotic, vancomycin, and Tazocin [piperacillin /tazobactam]) were: sepsis, allergic /allergic, intravenous, cannula. Most common terms related to **analgesics** (paracetamol, morphine, oxycodone, Oramorph [morphine sulphate elixir], fentanyl and tramadol) were: pain/pain relief, check, book, tablet, and theatre (Table 3).

**Table 3:** Most common drugs described in free text of incident reports (n = 72,390) and related terms based on concept linking

Drug	Freq.	Docs	Highly associated terms based on concept linking
insulin	10,086	4214	unit, time, level, morning, administer, evening, visit, nurse
antibiotic	6280	4313	sepsis, due, allergic, time, night, dose, ward, cannula
paracetamol	5449	3071	relief, pain, pain relief, child, hour, dose, orally, theatre
morphine	4194	2244	syringe, analgesia, pump, book, relief, pain, pain relief, check
saline	2676	1848	water, dilute, label, commence, check, arm, contrast, cannula, ctpa [CT pulmonary angiography]
oxycodone	2666	1521	morphine, balance, book, tablet, pain, bottle, check, immediate, prn
warfarin	2319	1151	valve, tinzaparin, INR, clinic, result, yellow, dose, patient warfarin
glucose	2138	1445	pump, saline, high, infusion, unit, level, commence, check, insulin
gentamicin	2098	1180	intravenous, benzylpenicillin, metronidazole, antibiotic, sepsis, due, hour, dose, level
chemotherapy	2052	1355	flush, bag, day, trial, unit, treatment, commence, first, line
amoxicillin	2031	1455	intravenous, oral, wrist, rash, reaction, metronidazole, allergy, antibiotic, allergic
fentanyl	1843	1193	anaesthetist, syringe, book, pain, find, old, check, patch, theatre
dalteparin	1829	1128	prophylactic, INR, pulmonary, sign, prophylaxis, warfarin, risk, unit, injection
potassium	1728	921	fluid, magnesium, pump, saline, high, infusion, level, commence
oramorph	1726	1145	morphine, amount, analgesia, book, relief, pain, pain relief, bottle, prn
furosemide	1568	984	run, heart, intravenous, rate, pump, hour, infusion
penicillin	1453	929	benzylpenicillin, wrist, rash, reaction, allergy, antibiotic, sepsis, allergic, know
intravenous antibiotic	1419	1224	midwife, baby, intravenous, cannulation, benzylpenicillin, antibiotic, sepsis, due, cannula
Clexane [enoxaparin]	1404	848	prophylactic, sign, warfarin, therapeutic Clexane [enoxaparin], treatment dose, therapeutic, injection
sodium	1401	1031	glucose, arterial, transducer, bag, fluid, potassium, phosphate, infusion, dilute
vancomycin	1364	751	difficile, continuous infusion, loading, intravenous, continuous, infusion, dose, level
dextrose	1233	777	protocol, bag, normal, fluid, potassium, saline, infusion, commence, insulin
tramadol	1230	703	cupboard, release, CD [controlled drugs] book, capsule, book, tablet, stock, immediate
midazolam	1207	727	draw, vial, fentanyl, unresponsive, morphine, syringe, book, care, end
enaxoparin	1201	826	treatment dose, prophylactic, sc, critical, INR, sign, prophylaxis, injection
anaesthetic	1114	819	record, immediately, post, case, surgery, theatre, list, surgeon
Tazocin [piperacillin / tezobactam]	1078	774	penicillin, intravenous, rash, reaction, allergy, antibiotic, sepsis, allergic
chloride	1068	747	flush, bag, fluid, potassium, saline, infusion, water, dilute, label
tinzaparin	1049	643	INR, therapeutic tinzaparin, treatment dose, therapeutic, warfarin, unit, injection
aspirin	879	582	chest pain, omeprazole, fondaparinex, Ramipril, ticagrelor, stroke, lansoprazole, clopidogrel

### Clusters

Data analysis produced 18 different clusters with RMSSD values of 0.069–0.134. The descriptive terms of the clusters typically included some drug names. For example, antibacterial drugs were found in multiple clusters. In cluster 3 (*n* = 1258 documents, 2% of all documents) descriptive terms included **penicillin and amoxicillin** (intravenous, allergy /allergic, reaction), in cluster 10 (*n* = 692, 1%) they included **chloramphenicol** (eye drop) and in cluster 16 (*n* = 3847, 5%) they included antibiotic (intravenous). Analgesics were also found in multiple clusters: clusters 1 (*n* = 1516, 2%) and 12 (*n* = 1816, 3%) both included **morphine** (with the terms such as patient-controlled analgesia, infusion, pump), cluster 4 (*n* = 1002, 1%) included **fentanyl** (patch) and **buprenorphine** (remove, find, pain), cluster 13 (*n* = 3969, 5%) included **oxycodone** (Table 4)

**Table 4:** Clusters (number and, % of incident reports within the cluster) and descriptive terms of medication administration incident reports (*n* = 72,390), and RMSSD† for testing the goodness of fit of clusters

Cluster	Descriptive terms^b^	No.	%	RMSSD^a^
1	syringe, driver, infusion, label, pump, run, **morphine**, line, commence, running	1516	2	0.093
2	infusion, hour, rate, pump, check, run, intravenous, start, running, stop	3317	5	0.102
3	allergy, **penicillin**, allergic, **amoxicillin**, reaction, intravenous, note, state, document, antibiotic	1258	2	0.077
4	patch, **fentanyl**, find, **buprenorphine**, ‘**fentanyl patch**’, apply, remove, date, pain, visit	1002	1	0.069
5	**insulin,** administer, morning, blood, unit, scale, diabetic, glucose, sugar, visit	3595	5	0.100
6	nurse, ward, staff, pharmacy, inform, night, doctor, discharge, pharmacist, home	15,709	22	0.126
7	find, date, bottle, box, **chemotherapy**, treatment, cupboard, pain, attend, book	8795	12	0.134
8	**warfarin**, dose, INR, chart, **dalteparin**, prescribe, day, doctor, sign, visit	962	1	0.075
9	**paracetamol**, patient, dose, intravenous, discharge, hour, home, pain, oral, medication	3455	5	0.113
10	drop, eye, eye drop, right left, **chloramphenicol**, bottle, pharmacy, attend, treatment	692	1	0.073
11	medication, patient, ward, administer, morning, sign, miss, wrong, pharmacy, transfer	5041	7	0.108
12	**morphine**, check, **sulphate**, pca, pain, prescription, control, book, oral, syringe	1816	3	0.098
13	patient, tablet, drug, check, **oxycodone**, find, book, release, control, cupboard	3969	5	0.114
14	injection, administer, scan, unit, contrast, **dalteparin**, cannula, extravasation, arm, attend	2632	4	0.115
15	dose, patient, prescribe, miss, administer, day, morning, night, sign, due	6964	10	0.115
16	intravenous, **antibiotic**, prescribe, fluid, intravenous antibiotic, oral, intravenous infusion, hour, **saline**	4884	7	0.115
17	bag, tpn, find, fluid, pharmacy, run, **saline**, line, start, running	2530	3	0.110
18	drug chart, drug, dose, prescribe, sign, day, note, morning, notice	4253	6	0.099
Total		72,390	100	

^a^ root mean square standard deviation

^b^ drugs are bolded

.

### Risk areas of medication administration

Based on the results of concept linking (Table 3), clustering (Table 4), and weights between identified mediations and highly associated terms (Additional file 1), the following risk areas were identified (with an example using incident reports):

1. Allergic reactions with antibacterial drugs
*“Received patient from ED [emergency department]. Viewed drug chart with transfer nurse and found patient was given amoxicillin despite having an allergic reaction to penicillin. Patient was closely monitored for signs of anaphylaxis and doctors were aware...”*
2. Intravenous administration of antibacterial drugs
*“Three doses intravenous antibiotics missed due to no venous access. Last dose IV [intravenous] Antibiotics given 24 hours earlier on 17 / 3 / 16 at 22:00. Patient states no one had tried to site a cannula since 10:00am. Patient is post-operative chronic congenital neutropaenic in Cubicle…”*
3. Fentanyl patches (removal old one before applying new)
*“On changing patient Fentanyl patch as per Drug Chart, I noted that there were two other fentanyl patches in situ x1 on Left arm, x1 on Right chest…”*
4. Checking and documenting of analgesic doses
*“Came on shift (12/10) went to give morphine for a patient. Was looking through the CD [controlled drugs] book to double check when he last was given it. noted that they had but 10mg -5mls out of the CD book. when he was only prescribed 5mg-2.5mls...”*
5. Checking doses of anticoagulants
*“Doctor re-prescribed warfarin dose after checking INR [international normalized ratio] level, stated patient was now on 4mg dose, 4mg warfarin given, went to sign drug card and realised pt [patient] had 3mg at 18:00, patient has now had 7mg warfarin…”*
6. Insulin doses and blood glucose
*“Morning dose of fast acting and long acting insulin missed. Patient has not received his breakfast yet at the time when morning medication was done. Informed patient that I will return to do his insulin when he gets his breakfast, however failed to return due to ward distractions. Mistake was noted at 12:00 when blood sugars was done before lunch and noted to be 23.”*
7. Administration of intravenous infusions, especially potassium, chloride, saline (sodium chloride 0.9%), sodium, glucose, dextrose
*“Patient has been administered the wrong medication. On the drug chart was prescribed normal saline 0.9 % with Potassium 40 mmol and patient was having Potassium Chloride 0.3% + Sodium Chloride 0.18% and Glucose 4 %. The prescription was signed and checked by day team who was looking after the patient…”*

## Discussion

As far as we are aware, this is the first study to extract information about medications from free text descriptions of medication administration incident reports, and to identify terms most frequently associated with risk. However, some previous studies have analysed NRLS medication safety incidents over 6 or 7 years period [18, 19], but those analyses were lacking the free text analysis about the involved medications.

### Implications for practice

Comparing our findings with high risk drug lists, many findings were similar, such as anti-thrombotics/ low molecular weight heparins /anticoagulants [2–4], insulin [2, 3], narcotics/opioids [2–4], parenteral nutrition, anaesthetics, and chemotherapeutics [2]. Anticoagulants, antibacterial drugs and opioids were also the most common drugs identified in a previous study that described medication administration incidents causing patient death [20]. These similarities were interesting, especially because most (86%) of the incidents in the present study were not reported to have caused patient harm, in contrast to all incidents reported to NRLS as occurring between October 2017 and September 2018 for which the corresponding figure was 75% [21]. One possible reason for this lower level of reported harm in the present study is that medication administration incidents might be more easily witnessed and near misses therefore more likely to be reported.

The risks areas of medication administration related to specific drugs were identified. Special attention should be paid to avoiding allergic reactions with antibacterial drugs by verifying patient allergies before administration of drugs and by monitoring patients’ symptoms carefully. Additional strategies to address problems with patients’ documented allergies include adding clear and visible prompts, listing patient allergies and a description of the reaction, and making the allergy reaction selection mandatory in organisations using electronic prescribing [22]. Patients should also be aware of these risks and report signs of allergic reactions.

More attention should also be paid to intravenous administration especially related to antibacterial drugs, but also infusions such as potassium, chloride, saline (sodium chloride 0.9%), dextrose. Intravenous administration is a complex process and errors occurring at any stage can cause harmful patient outcomes [23], with a higher risk than other medication administrations [24]. More attention should also be paid to removal of fentanyl (and other) transdermal patches when applying a new patch, checking and documenting of doses of analgesics, anticoagulants and insulin. Bar-code medication administration systems may also decrease the potential of these types of errors [25].

As incident reports are valuable data source for identifying risk areas of medication safety and plentiful data has already accumulated in organizations, organisations should use text mining or similar methods within organisations, to look at their own incident report data for identifying these risk areas. This is important due to limitations in the quality of incident reports, such as underreporting and indeterminate data, as well as inaccuracies in reporting that jeopardize the overall usefulness of these data [26]. In addition, free text descriptions are a potentially very useful part of incident reports, but manually identifying common risk areas with big data sets can be very challenging. In the future, it is possible to implement real-time monitoring systems to alert for trends in incidence reporting. Possible other implications could be comparisons between point-of-care and monitoring of impact after changes to current processes.

### Implications for research

Risk areas identified in the present study should be compared using similar analytical approaches on other data sets, such as primary care data. In addition, future work could focus on analysing the risk areas of the most harmful errors, such as fatal medication administration errors [20]. The findings from this study can also be used to form hypotheses for further study. Text mining methodology should be developed further to produce more effective mining of essential characteristics and factors contributing to incidents from free text descriptions of incident reports and similar text-based data sets.

### Strengths and limitations

The SAS text mining application was useful for analysing this large dataset that included free text from over 70,000 incident reports and helpful in identifying the concept links between terms and for clustering the data. The credibility of text mining has previously been recognized and tested [5] and its accuracy, sensitivity and specificity shown to be high when compared with manual analysis [27]. One of the most significant advantages of SAS text-miner software is its computational speed in clustering a large volume of textual data within a short time [5], e.g. processing of tens of thousands of documents will take only minutes, when manual inspection would take months. Most of the free text descriptions in incident reports are short, so one challenge in clustering is the high dimensionality and sparsity of the term-document matrix, but singular value decomposition (SVD) reduces the dimensionality by transforming the term-document matrix into a lower dimension [5].

Additionally, the analyses required the researchers to make some subjective decisions, such as interpreting the results based on clustering and concept linking [5, 16]. One challenge is providing a description of the contents of the clusters. Short cluster names only provide a partial description of the content, possibly omitting important characteristics [16]. In addition, when terms are clustered together with a certain strength of association it does not necessary capture the whole meaning, for example, incidents where a drug happens to be mentioned in relation to the incident but was not the drug or only drug involved in the incident. Some drugs could be mentioned more than once in the free-text. For example, there were 4214 documents where insulin was mentioned in free text but only 2577 times in ‘Approved Drug name’ field. One explanation for this is that a specific drug name, such as Actrapid, was used in ‘Approved Drug name’ field, but in the free text description of the incident, the term insulin was used instead of using specific name. In addition, 14% (*n* = 10,414) of the incident reports lacked the named drug in the ‘Approved Drug name’ field (field was empty), and in over 400 reports the word ‘none’ was written and in over 2500 incidents ‘no drug given’. The results based on these analyses are therefore only indicative but give a direction of travel for future studies. The value of this methods of analysis is its ability to identify specific themes within a large dataset that would be impossible to obtain manually.

In addition, combining synonyms was challenging without understanding the original meaning of the word. Many words can be either a verb, adjective, noun, or have multiple meanings due to the flexibility of language with the same meaning expressed in different ways [28]. Some words were also written in multiple ways including some with typing errors. However, most typing errors and misspellings were automatically combined correctly, for example, the term insulin could be misspelled as isulin, insuline, inslin, insuliln, inslulin, insuin, insuln, insuling, insulkin, insuln, insilin, inulin, insulie, insulan, inzulin, insullin, inuslin, insulnin, insuilin, isuline, insluin, inuslin, insukin, insuli, or insulins. However, it remains possible that the software missed some misspelled drug names, thus the results are only indicative.

Incident report data suffers from under-reporting and the quality of reports may vary in terms of detail and accuracy [26, 29]. These issues may introduce biases. For example, many of the free text descriptions were quite short which may lead to inadequate information and weak linkage to particular clusters [5]. In addition, free text descriptions do not necessarily list all involved medications / drug names, thus limiting the evidence produced.

## Conclusion

This analysis suggests that interventions to increase medication administration safety should focus on checking patient allergies and medication doses, especially for intravenous and transdermal medication, as well as taking action to avoid dose omissions. High risk medications include insulin, analgesics, antibacterial drugs, anticoagulants, and potassium chloride. Text mining may be useful for analysing large free text datasets and should be developed further to allow more effective mining of essential characteristics and factors contributing to medication incidents.

## Supplementary information

**Additional file 1.** Weights between most common medications and highly associated terms based on clustering and concept linking.

## Data Availability

The data that support the findings of this study are available from NRLS/ NHS Improvement but restrictions apply to the availability of these data, which were used under license for the current study, and so are not publicly available. Data are however available from the authors upon reasonable request and with permission of NRLS / NHS Improvement.

## Abbreviations

INR: International Normalized Ratio
ISMP: Institute for Safe Medication Practices (US)
NLP: Natural language processing
NPSA: National Patient Safety Agency (England and Wales)
NRLS: National Reporting & Learning System
NSAIDS: Nonsteroidal anti-inflammatory drugs
RMSSD: Root mean square standard deviation
