# Supplementary material for: Identifying risks areas related to medication administrations - text mining analysis using free-text descriptions of incident reports
Source: BMC Health Serv Res. 2019 Nov 4;19:791. doi: 10.1186/s12913-019-4597-9 (PMC6829803; doi:10.1186/s12913-019-4597-9)
Supplement: Supplementary file 1 — Additional file 1. Weights between most common medications and highly associated terms based on clustering and concept linking. [file 12913_2019_4597_MOESM1_ESM.docx]

Additional file 1. Weights between most common medications and highly associated terms based on clustering and concept linking

| **Medications based on clustering** | **Highly associated terms** | **Frequency of terms with specific corpus / weights** |
| --- | --- | --- |
| fentanyl | Clustering: patch, find, buprenorphine, ‘fentanyl patch', apply, remove, date, pain, visit  Concept linking: anaesthetist, syringe, book, pain, find, old, check, patch, theatre | buprenorphine n=34 / 0.601  date n=36 / 0.550  visit n=47/ 0.525  anaesthetist n=115 / 0.465  old n=62 / 0.430  apply n=98 / 0.424  remove n=101 / 0.382  theatre n=152 / 0.371  syringe n=222 / 0.335  book n=166 / 0.331  pain n=315 / 0.273  ‘fentanyl patch' n=283 / 0.242  find n=276 / 0.234  check n=598 0.182  patch n=953/ 0.173 |
| fentanyl patch | Clustering: patch, fentanyl, find, buprenorphine, apply, remove, date, pain, visit | visit n=99 / 0.445  date n=117 / 0.401  old n=111 / 0.397  remove n= 240 / 0.314  apply n=284 / 0.311  buprenorphine n=295 / 0.295  pain n=426 / 0.277  find n=447 / 0.206  check n=721 / 0.188  patch n=2395 / 0.102  fentanyl n=1800 / 0.084 |
| morphine | Clustering: prescribe, allergy, penicillin, allergic, intravenous, reaction, amoxicillin, sulphate, pca, syringe, driver, infusion, pump, label, run, morphine, line, running, visit  Concept linking: syringe, analgesia, pump, book, relief, pain, pain relief, check | reaction n=43 / 0.558  allergic n=43 / 0.543  visit n= 48 / 0.525  line n=80 / 0.517  allergy n=84 / 0.491  relief n=66 / 0.470  running n=88/ 0.445  run n=116 / 0.433  analgesia n=151 / 0.39  driver n=312 / 0.387  infusion n= 474/ 0.308  pump n=275 / 0.356  label n= 162 / 0.383  book n=318 / 0.293  intravenous n=508 / 0.272  pca n=763 / 0.263  syringe n=754 / 0.258  pain n=675 / 0.242  sulphate n=661 / 0.202  check n= 969 / 0.168  prescribe n=958 / 0.16  penicillin n=12 / 0.00  amoxicillin n=11/ 0.00 |
| oxycodone | Clustering: drug, check, book, control, cupboard, find, release, sign, cd book  Concept linking: morphine, balance, book, tablet, pain, bottle, check, immediate, prn | balance n= 113 / 0.443  bottle n= 154 / 0.410  cd book n= 146 / 0.345  morphine n= 252 / 0.352  cupboard n=219 / 0.303  prn n= 249 / 0.288  pain n= 297 / 0.289  sign n=308 / 0.277  book n=321 / 0.257  immediate n=268 / 0.279  control n=282 / 0.272  find n=258 / 0.268  book n=321 / 0.257  release n= 599 / 0.229  tablet n= 654 / 0.218  check n=658 / 0.171  drug n=846 / 0.161 |
| sulphate | Clustering: morphine, prescribe, allergy, penicillin, allergic, intravenous, reaction, amoxicillin | morphine n=1485 / 0.069  intravenous n=140 / 0.397  prescribe n=380 / 0.177  allergy n=8 / 0.000  penicillin n=1 / 0.000  allergic n=3 / 0.000  reaction n=4 / 0.000  amoxicillin n=2 / 0.000 |
| antibiotic | Clustering: intravenous, dose, prescribe, intravenous antibiotic, oral, miss, due hour, amoxicillin  Concept linking: sepsis, due, allergic, time, night, dose, ward, cannula | sepsis n=73/ 0.445  allergic n=92 / 0.424  cannula n=121 / 0.422  amoxicillin n=212 / 0.33  hour n=250 / 0.295  oral n=269 / 0.293  intravenous antibiotic n=265 / 0.274  miss n=6 / 0.27  time n=322 / 0.255  night n=363 / 0.254  ward n=702 / 0.209  intravenous n=1141 / 0.129  dose n=1367 / 0.127  prescribe n=699 / 0.17 |
| penicillin | Clustering: morphine, prescribe, allergy, allergic, intravenous, reaction, amoxicillin, sulphate, pca  Concept linking: benzylpenicillin, wrist, rash, reaction, allergy, antibiotic, sepsis, allergic, know | sepsis n=52 / 0.458  wrist n=47 / 0.445  benzylpenicillin n=85 / 0.420  know n=103 / 0.331  rash n=125 / 0.322  antibiotic n=306 / 0.237  reaction n=228 / 0.228  prescribe n=447 / 0.137  allergy n=842 / 0.102  allergic n=488 / 0.119  intravenous n=411 / 0.171  amoxicillin n=405 / 0.174  morphine n=7 / 0.000  sulphate n=1 / 0.000  pca n=0 / 0.000 |
| amoxicillin | Clustering: morphine, prescribe, allergy, penicillin, allergic, intravenous, reaction, sulphate, pca, antibiotic, dose, intravenous antibiotic, oral, miss, due hour  Concept linking: intravenous, oral, wrist, rash, reaction, metronidazole, allergy, antibiotic, allergic | wrist n=11 / 0.611  rash n=30 / 0.504  intravenous antibiotic n=35 / 0.443  miss n=39 / 0.443  reaction n=53 / 0.431  hour n=48 / 0.416  metronidazole n=62 / 0.413  allergic n=81 / 0.327  oral n=94 / 0.318  penicillin n=133 / 0.306  allergy n=155 / 0.298  antibiotic n=200 / 0.230  intravenous n=304 / 0.173  dose n=402 / 0.179  prescribe n=226 / 0.185  morphine n=2 / 0.000  sulphate n=1 / 0.000  pca n=0 / 0.000 |
| warfarin | Clustering: dose, prescribe, inr, dalteparin, sign, unit, patient, day, doctor  Concept linking: valve, tinzaparin, inr, clinic, result, yellow, dose, patient warfarin | valve n=31 / 0.564  tinzaparin n=118 / 0.468  yellow n=60 / 0.468  unit n=83 / 0.452  clinic n=111 / 0.414  dalteparin n=182 / 0.398  doctor n=289 / 0.274  sign n=248 / 0.259  inr n=502 / 0.212  day n=415 / 0.209  dose n=955 / 0.128  prescribe n=572 / 0.158  patient n=2554 / 0.058 |
| dalteparin | Clustering: warfarin, dose, prescribe, inr, sign, unit, patient, day, administer, inform, doctor, error, blood, pain, theatre, visit  Concept linking: prophylactic, inr, pulmonary, sign, prophylaxis, warfarin, risk, unit, injection | pulmonary n=21 / 0.562  visit n=30 / 0.534  blood n=35 / 0.508  prophylaxis n=34 / 0.493  inr n=62 / 0.490  prophylactic n=45 / 0.465  risk n=73 / 0.419  warfarin n=118 / 0.406  error n=89 / 0.383  inform n=113 / 0.369  doctor n=152 / 0.325  injection n=209 / 0.308  sign n=264 / 0.237  unit n=320 / 0.229  patient n=1598 / 0.079  day n=280 / 0.231  administer n=314 / 0.214  dose n=731 / 0.137  prescribe n=413 / 0.16  theatre n=13 / 0.000 |
| insulin | Clustering: administer, blood, morning, unit, glucose, diabetic, sugar, visit, evening  Concept linking: unit, time, level, morning, administer, evening, visit, nurse | level n=427 / 0.326  visit n=540 / 0.300  sugar n=614 / 0.282  evening n=658 / 0.265  glucose n=1006 / 0.245  diabetic n=722 / 0.240  unit n=1671 / 0.206  morning n=1384 / 0.179  administer n=1688 / 0.17  blood n=1628 / 0.185  nurse n=2604 / 0.152 |
| glucose | Clustering: administer, blood, morning, unit, insulin, diabetic, sugar, visit, evening  Concept linking: pump, saline, high, infusion, unit, level, commence, check, insulin | visit n=105 / 0.431  evening n=123 / 0.399  sugar n=153 / 0.381  pump n=190/ 0.359  high n=125 / 0.353  diabetic n=222 / 0.296  unit n=349 / 0.283  morning n=258 / 0.281  saline n=310 / 0.261  level n=382 / 0.240  administer n=427 / 0.239  infusion n=766 / 0.214  check n=699 / 0.170  insulin n=1858 / 0.144  blood n=1161 / 0.118 |
| saline | Clustering: cannula, flush, line, intravenous, infusion, pain, normal, contrast, scan  Concept linking: water, dilute, label, commence, check, arm, contrast, cannula, ctpa | ctpa n=30 / 0.566  water n=47 / 0.509  scan n=197 / 0.428  contrast n=197 / 0.410  dilute n=124 / 0.404  arm n=135 / 0.403  pain n=147 / 0.387  label n=215 / 0.342  commence n=223 / 0.314  line n=334 / 0.310  cannula n=482 / 0.285  flush n=547 / 0.234  intravenous n=941 / 0.171  infusion n=1049 / 0.196  check n=784 / 0.172  normal n=1027 / 0.118 |
| paracetamol | Clustering: patient, dose, intravenous, hour, cefuroxime, pain, theatre, oral, prescribe  Concept linking: relief, pain, pain relief, child, hour, dose, orally, theatre | theatre n=450 / 0.309  pain n=666 / 0.258  oral n=373 / 0.288  orally n=86 / 0.450  pain relief n=116 / 0.43  relief n=134 / 0.416  child n=190 / 0.430  patient n=5931 / 0.022  dose n=2168 / 0.134  intravenous n=1316 / 0.182  cefuroxime n=1142 / 0.190  prescribe n=1252 / 0.159 |
